# Plasma Levels of Acylation-Stimulating Protein Are Strongly Predicted by Waist/Hip Ratio and Correlate with Decreased LDL Size in Men

**DOI:** 10.1155/2013/342802

**Published:** 2013-02-12

**Authors:** Jumana Saleh, Rabab A. Wahab, Hatem Farhan, Issa Al-Amri, Katherine Cianflone

**Affiliations:** ^1^Biochemistry Department, College of Medicine and Health Sciences, Sultan Qaboos University, P.O. Box 35, Muscat, Oman; ^2^Integrated Sciences Division, College of Health Sciences, University of Bahrain, P.O. Box 32038, Manama, Bahrain; ^3^College of Medicine and Health Sciences, Sultan Qaboos University, P.O. Box 35, Muscat, Oman; ^4^Centre de Recherche Hôpital Laval, Université Laval, Y2186, 2725 Chemin Ste-Foy, Québec, QC, Canada G1V 4G5

## Abstract

The association of abdominal obesity with cardiovascular risk is often linked to altered secretion of adipose-derived factors and an abnormal lipid profile including formation of atherogenic small dense low density lipoprotein particles (sdLDL). Acylation-stimulating protein (ASP) is an adipose-derived hormone that exhibits potent lipogenic effects. Plasma ASP levels increase in obesity; however, the association of ASP levels with body fat distribution is not yet established, and no study to date has investigated the association of ASP with LDL size. In this study, we examined the association of ASP levels with abdominal obesity measures and the lipid profile including LDL size in 83 men with a wide range of abdominal girths. Regression analysis showed that waist/hip ratio was the main predictor of ASP levels (**β** = 0.52, *P* < 0.0001), significantly followed by decreased LDL size. BMI and TG levels, although positively correlated with ASP levels, were excluded as significant predictors in regression analysis. No correlation was found with LDL-C or apoB levels. ASP levels were 62.5% higher in abdominally obese compared to nonobese men. Waist/hip ratio presenting as the main predictor of ASP levels, suggests increased ASP production by abdominal fat which, as proposed previously, may result from resistance to ASP function causing delayed TG clearance and subsequent formation of atherogenic sdLDL.

## 1. Introduction

The link between abdominal (omental) obesity and cardiovascular risk is well recognized. The dyslipidemic profile of abdominal obesity, and its association with atherogenic risk [1, 2] is attributed to insulin resistance and altered secretion of fat derived factors including fatty acids and adipokines such as adiponectin, leptin, interleukin-6, and tumor necrosis factor-alpha [3].

 Acylation-stimulating protein (ASP) is another adipokine that was isolated based on its function as a potent fat storage factor. ASP was shown to be comparable to insulin in its fat storing stimulatory effect [4]. Abundant *in vivo* and *in vitro* evidence linked ASP function to enhanced triglyceride clearance by promoting fatty acid trapping and fat storage in adipocytes by activating the rate-limiting enzyme for TG synthesis, diacylglycerol acyl transferase [5]. Insulin, on the other hand, activates lipoprotein lipase releasing fatty acids for uptake by adipocytes and inhibits hormone-sensitive lipase which catalyzes fat hydrolysis from adipocytes. ASP acts in a manner that is independent but additive to insulin [6]. ASP levels were shown to increase in obese and hyperlipidemic subjects [5, 7] and decrease with weight loss and fasting [5, 8].

The functional receptor, C5L2, was recently identified as the ASP receptor [9]. Subjects with a variant of the C5L2 receptor demonstrated marked reduction in ASP binding associated with increased plasma TG and ASP levels, and a significant reduction in ASP stimulated TG synthesis and glucose transport suggestive of receptor downregulation [10]. Several studies demonstrated that increased ASP levels positively correlated with increased TG levels, while a weaker correlation was found with total and LDL cholesterol or apoB levels [11, 12]. These findings were explained by resistance to ASP function. Several studies have supported the concept of ASP resistance [13]. In hyperlipidemic subjects, ASP resistance was suggested to contribute to increased TG levels as a result of delayed ASP-mediated TG clearance [5, 11, 14, 15].

Increased TG and low HDL-C levels, as a consequence of delayed TG clearance, are important determinants of the dyslipidemic profile associated with abdominal obesity [16], and the TG/HDL-C ratio is a recognized predictor of LDL size variations in serum [17, 18]. Since ASP is largely implicated in enhancing fat storage and plasma TG clearance, we investigated the association of plasma ASP levels with markers of abdominal obesity and its related metabolic profile including waist/hip ratio, TG, HDL-C, and other lipid profile parameters including LDL particle size which has not been investigated previously.

## 2. Methods

### 2.1. Subjects and Study Design

A cross-sectional association study was designed. A sample of 83 Omani males was included in this study (average age 41.8 ± 12.1 years). The subjects fasted overnight before blood samples were collected. The subjects included had a wide range of abdominal girths as indicated by waist/hip ratio: 0.75–1.34. In order to compare differences in plasma ASP levels between abdominally obese and nonobese men, abdominally obese subjects were categorized as having a waist to hip ratio >0.95. This cutoff point for high waist/hip ratio was selected according to the American Heart Association to define high risk for cardiovascular disease in men [19–21]. The study was approved by Sultan Qaboos University ethical committee. Informed consent forms were filled out by all subjects participating in the study.

### 2.2. Analysis

Fasting blood samples were collected in plain tubes with no anticoagulant for lipid measurements and EDTA tubes for ASP measurements. The collection was performed in the morning, and samples were put on ice and immediately centrifuged. The serum and plasma were stored at −80°C until analysis. All samples were analyzed for the fasting lipid profile, including TG, total cholesterol (total-C), (LDL-C), (HDL-C), apolipoprotein apoB, and apoA1. Analysis was performed using an automated clinical chemistry analyzer CX 7 Super Clinical System (SYNCHRON) based on enzymatic colorimetric assays for lipid parameters: TG, total-C, HDL-C, and LDL-C. Analysis of samples for apoA and apoB was based on immunoturbidimetry of antigen antibody reactions with the sample apoprotein detected as antigen by IMMAGE Immunochemistry Systems. Both analyzers are products of Beckman Coulter, Inc. (Fullerton, CA).

### 2.3. Sandwich Enzyme-Linked Immunosorbent Assay (ELISA) for ASP Determination

Human blood EDTA samples were centrifuged, the plasma was separated, and ASP was assayed immediately by a sandwich ELISA using a monoclonal antibody as capture antibody and a polyclonal antibody as detecting antibody, as described in detail previously [22].

### 2.4. Nondenaturing Gradient Gel Electrophoresis to Measure LDL Size

Nondenaturing 3% to 16% polyacrylamide gradient gel electrophoresis (PAGGE) was performed as described previously [23]. Briefly, LDL particle size was determined on 16 cm × 16 cm polyacrylamide gradient gels prepared in our laboratory. Serum samples were adjusted to 40% sucrose and 0.05% bromophenol blue. 15 *μ*L were applied to the gel. Potentials were set at 125 V (15 min), 200 V (4 hr), and 300 V (2 hr). Gels were fixed and stained for lipids in a solution containing Oil Red O in 60% ethanol at 50°C for 24 hr and destained in acetic acid (5%) [23]. The gels were placed on filter paper and dried with gel drying system (BIO-RAD) for one 45 minutes at 80°C. The drying was achieved using double stage vacuum pump (Fisons, UK). Dried gels were photographed with a Bioimaging System (Chemi Genius^2^) using Gene-Snap program, and band densities were analyzed using Gene-Tools program. The program allows measurement of the migration of the bands in the gel by densitometry. LDL size was extrapolated from the relative migration of three LDL control samples and lipoprotein little a Lp (a) that were purified by electroelution for which particle size was measured by electron microscopy. The control samples were run alongside the samples and stained with Oil Red O. LDL size measurements were verified from the relative migration of protein standard samples (High Molecular Weight Native Marker Kit from GE Healthcare NJ) stained by Coommasie Blue.

### 2.5. Statistical Analysis

Bivariate correlations between different parameters were examined by analysis using Pearson coefficients. Spearman correlation coefficients were used for parameters with skewed distributions. Step-wise multiple linear regression analysis was performed to determine factors that significantly explain variations in fasting plasma ASP levels. ASP levels were expressed as mean ± standard error. The means were compared by independent sample Student's *t*-test. Levene's test was used to test equality of variances. Significance was set at *P* < 0.05. Analysis was computer assisted using the SPSS/PC statistical program (version 13.0 for Windows; SPSS, Inc., Chicago, IL).

## 3. Results

### 3.1. Correlation Analysis

**Figure 1:**
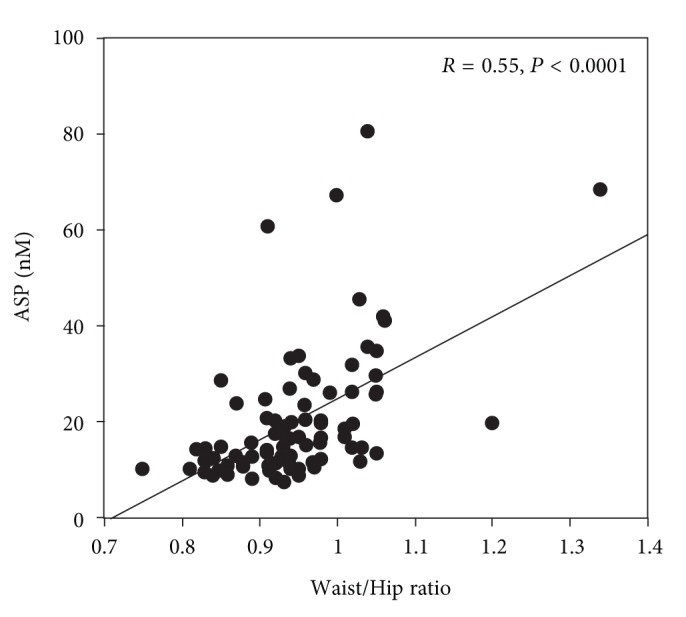
A scatter plot representing the correlation of ASP with waist/hip ratio. Individual data and regression lines are shown. The slopes of the regression lines are all significant (controls: *r* = 0.55, *P* < 0.0001).

Table 1 shows the correlation of ASP with all measured anthropometric and lipid parameters using bivariate analysis. Significant positive correlations were found for ASP with waist/hip ratio, BMI, age, TG, and very low density cholesterol (VLDL-C) levels. No correlation was found with total cholesterol, LDL-C, HDL-C, apoB levels, or glucose levels.  Figure 1 shows a scatter plot representing the correlation of ASP with waist/hip ratio for all the subjects. A significant negative correlation (*r* = −0.25, *P* = 0.025) was found between fasting plasma ASP levels and LDL size, a result supported by a significant correlation of ASP with TG/HDL-C (*r* = 0.28, *P* = 0.01) which is a recognized predictor of LDL size [24].

### 3.2. Stepwise Multiple Regression Analysis

A stepwise multiple linear regression model was set to determine the parameters that significantly predict ASP variations in this study. ASP was included as the dependent variable. Independent variables included waist/hip ratio, BMI, age and lipid parameters: TG, total cholesterol, LDL-C, HDL-C, apoA1, apoB, and LDL size. Waist/hip ratio entered the model as the highest significant predictor (*β* = 0.52, *P* < 0.0001) followed by LDL size (*β* = −0.22, *P* = 0.02). Waist/hip ratio explained 26.3% variation in ASP levels (as determined by the adjusted *R* ^2^), followed by LDL size where both parameters determined 30% variation in ASP levels. BMI age, and all measured lipid and apoprotein parameters were excluded as nonsignificant predictors of ASP levels. 

Plasma ASP levels, lipid, and anthropometric profiles were investigated for all subjects. 

**Figure 2:**
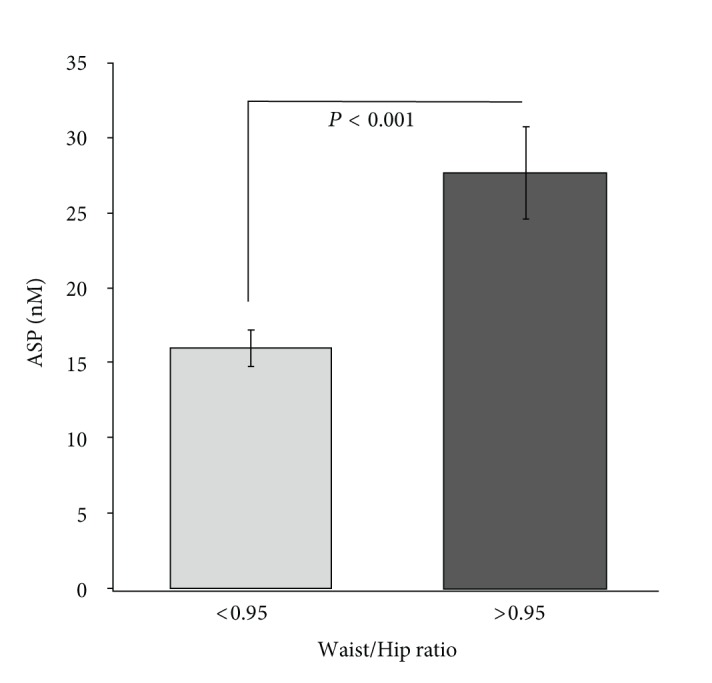
Fasting acylation-stimulating protein (ASP) levels are shown for abdominally obese subjects (waist/hip ratio > 0.95, *n* = 35) compared to nonobese subjects (waist/hip ratio < 0.95, *n* = 48). Data is shown as average ± S.E.M. Groups were compared by independent sample *t*-test using Levene's test for equality of variances.

Figure 2 shows differences in ASP levels between abdominally obese (W/H ≥ 0.95, *n* = 35) and nonobese (W/H < 0.95, *n* = 48) subjects. Abdominally obese subjects had 62.5% increase in plasma ASP levels (*P* < 0.001) compared to nonobese subjects.

## 4. Discussion

Metabolic alterations that accompany intraabdominal fat accumulation typically involve increased TG levels, low HDL-C levels (increased TG/HDL-C ratio), formation of small dense LDL particles, and normal LDL-C levels [25]. Results in this study notably link plasma ASP levels to the metabolic profile associated with abdominal obesity. ASP levels were shown to be higher in males with abdominal obesity compared to nonobese subjects. ASP levels showed a significant positive correlation with waist/hip ratio, TG levels, and TG/HDL ratio and negatively correlated with HDL-C levels and LDL size. Consistent with previous studies, there was no correlation of ASP with LDL-C or apoprotein levels [11, 12, 26]. 

In previous studies, a significant positive association between ASP and TG levels independent of BMI was shown; however, waist/hip ratio and LDL size were not included as independent parameters in their analysis [11, 14, 27, 28]. In this study, a significant positive association was found for plasma ASP with TG levels however, adding waist/hip ratio and LDL size to the regression model excluded both plasma TG and BMI as significant predictors of ASP levels. Waist/hip ratio alone predicted 26.3% increase in ASP levels, followed by LDL size together with significantly predicting 30% variation in ASP levels. These marked variations suggest that waist/hip ratio, an abdominal obesity marker, surpasses the effect of plasma TG levels and all measured parameters in this study as a determinant of plasma ASP variation.

In order to explain increased ASP levels in association with abdominal obesity, we highlight “metabolic resistance” as an important feature that may be shared between insulin and ASP. It is well established that insulin resistance, reflected in increased plasma insulin levels, is an underlying feature of abdominal obesity, and a major component of the metabolic syndrome [25]. *In vitro* and *in vivo* evidence support that the concept of ASP resistance may exist as well. ASP resistance, in association with increased ASP and TG levels, was introduced in several studies [5, 10, 11, 13–15]. Increased ASP levels in abdominal obesity may result from decreased response of intra-abdominal (omental) fat to ASP function. This is supported by studies showing that ASP binding affinity was markedly lower in plasma membrane extracts from omental compared to subcutaneous adipose tissue, and lower in male compared to female adipose tissue [29]. Furthermore, functional studies showed that ASP-mediated triglyceride synthesis was markedly lower in omental adipocytes compared to subcutaneous adipocytes [30]. *In vivo* studies showed a decreased response to ASP-mediated triglyceride clearance in males compared to females in humans and mice [31].

**Figure 3:**
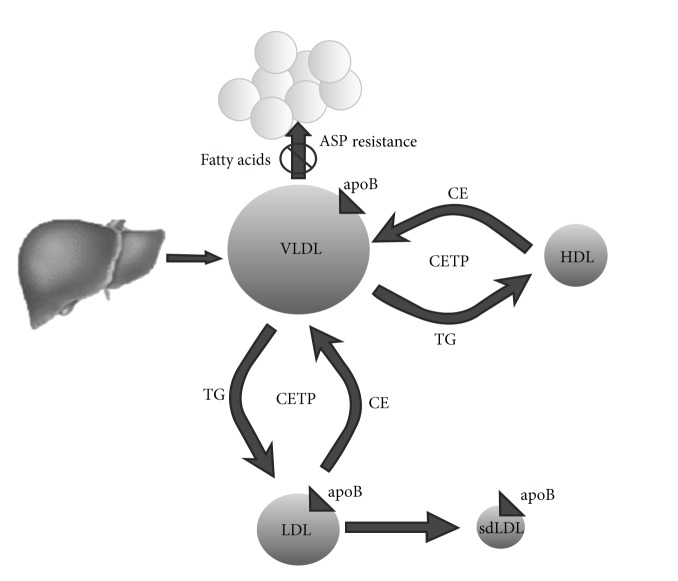
A hypothetical scheme suggesting that increased circulating triglyceride rich VLDL particles due to decreased clearance may accelerate metabolic events involving cholesterol ester transfer protein (CETP) engaged in conversion of existing circulating VLDL into LDL and further conversion into small dense LDL (sdLDL).

Findings in this study suggest that, with accumulation of omental fat in males, ASP resistance may develop which is reflected in increased plasma ASP levels and decreased plasma TG clearance. Accumulation of plasma TG rich-VLDL levels may therefore accelerate metabolic events that normally catalyze the conversion of VLDL particles into small dense LDL particles in association with decreased HDL-C levels [25, 32, 33] (Figure 3). These findings are consistent with adverse metabolic events related to abdominal obesity.

The association of ASP with abdominal obesity is not yet established. Limited studies investigated the correlation of ASP with waist/hip ratio. One study showed no significant association between ASP and waist/hip ratio in females [34]. Another study showed a significant positive association in a small sample of males [12]. However, predictors of ASP levels were not reported in these studies.

To our knowledge, this is the first study highlighting that increased waist/hip ratio, in association with decreased LDL size, significantly determine plasma ASP levels, which may link ASP to atherogenic metabolic events in abdominally obese males.

In conclusion, elevated ASP levels in abdominal obesity, strongly predicted by waist/hip ratio, suggest that ASP production by abdominal fat may be increased as a result of ASP resistance, a concept proposed in previous studies [5, 11, 13–15]. ASP resistance may contribute to lipid alterations that encourage the formation of atherogenic small dense LDL particles, and therefore increase the risk of coronary artery disease (CAD) in abdominally obese subjects.

These findings highlight ASP as another adipokine metabolic marker [35] that may contribute to further understanding of the pathophysiology of abdominal obesity and its link to cardiovascular risk in men.

## Figures and Tables

**Table 1:** Bivariate correlations of ASP with anthropometric and lipid parameters.

Parameter	* R *	*P* value
W/H	0.55	<0.0001
BMI	0.264	0.017
TG	0.23	0.037
Chol	−0.04	0.86
LDL-C	−0.038	0.73
HDL-C	−0.21	0.05
VLDL-C	0.23	0.03
ApoB	0.087	0.44
ApoA1	−0.204	0.06
TG/HDL-C	0.283	0.01
LDL-C/HDL-C	0.13	0.24
LDL size	−0.25	0.025

*P* < 0.05 = significant, two tailed.

## List of Abbreviation

ASP: Acylation-stimulating protein
TG: Triglycerides
VLDL-C: Very low density lipoprotein cholesterol
LDL-C: Low density lipoprotein cholesterol
HDL-C: High density lipoprotein cholesterol
sdLDL: Small dense low density lipoprotein
apoB: Apolipoprotein B
apoA1: Apolipoprotein A1
Waist/hip ratio: Waist to hip ratio
CETP: Cholesterol ester transfer protein
ELISA: Enzyme-linked immunosorbent assay
S.E.M: Standard error of the mean.
